# Large future genetic diversity losses are predicted from conservation indicators even with habitat protection

**DOI:** 10.1073/pnas.2514371123

**Published:** 2026-03-26

**Authors:** Kristy S. Mualim, Jeffrey P. Spence, Clemens Weiß, Oliver Selmoni, Meixi Lin, Moises Exposito-Alonso

**Affiliations:** ^a^Department of Plant Biology, Carnegie Institution for Science, Stanford, CA 94305; ^b^Department of Biology, Stanford University, Stanford, CA 94305; ^c^Department of Integrative Biology, University of California Berkeley, Berkeley, CA 94720; ^d^Department of Genetics, Stanford University, Stanford, CA 94305; ^e^Institute for Human Genetics, University of California, San Francisco, CA 94158; ^f^Department of Epidemiology & Biostatistics, University of California, San Francisco, CA 94158; ^g^Stanford Cancer Institute, Stanford University, Stanford, CA 94305; ^h^Department of Global Ecology, Carnegie Institution for Science, Stanford, CA 94305; ^i^HHMI, University of California Berkeley, Berkeley, CA 94720

**Keywords:** genetic diversity, habitat destruction, continuous space simulations

## Abstract

Genetic diversity within species underpins evolutionary adaptation and has recently been included as a target for protection in the United Nations’ Global Biodiversity Framework (GBF). Yet, we lack mathematical tools to estimate past genetic diversity loss across species—or predict future losses—based on demographic proxies used in conversation policy. To fill this gap, we developed a spatiotemporal framework to predict the dynamics of genetic diversity under realistic habitat change scenarios, calibrated with population-scale genomic data from 29 plant and animal species. To estimate how much genetic diversity has already been lost, we analyzed habitat area and population size losses for 4,611 species from the last five decades, using data from the Living Planet Index, the Red List, and new GBF indicators. We estimate that species have already lost 1 to 13% of π genetic diversity. Furthermore, we predict that genetic diversity losses lag behind population and habitat area declines, such that an average of 6 to 45% of genetic diversity will be lost under different scenarios even if population sizes or habitats do not decline further. Our results highlight that safeguarding existing habitats is insufficient to maintain the genetic health of species, and that genetic monitoring from proxy indicators will only detect major genetic diversity losses after it is already too late.

## Significance

Genetic diversity is crucial for both species adaptation and survival. Recently, it has been included as a target for protection in the United Nations’ Global Biodiversity Framework. However, we lack scalable predictive methods to quantify current and future losses of genetic diversity across species. Here, we develop a spatiotemporal predictive framework calibrated with high-quality genome-wide data from 29 plant and animal species to quantify genetic biodiversity at global scales. Our model predicts genetic diversity may have already declined beyond the preliminary UN targets of maintaining 90% of species’ genetic diversity and will continue declining over time, even with habitat protection.

Genetic diversity dictates a species’ ability to adapt to new environmental conditions (1) and was chosen as one of three key dimensions of biodiversity at the 1992 United Nations Convention of Biological Diversity (UN CBD) (2). Yet, genetic diversity remains largely overlooked in conservation practice, conservation targets, monitoring frameworks, and action plans. In 2022, the Kunming-Montreal UN CBD finally ratified a Global Biodiversity Framework (GBF) (CBD/COP/DEC/15/4, CBD/COP/DEC/15/5) that explicitly aims to “*maintain and restore the genetic diversity within and between populations of native, wild and domesticated species to maintain their adaptive potential*” (3). With increased political will and excitement around genetic diversity policy, it is imperative to develop the necessary tools to empower nations in achieving genetic diversity goals.

Under anthropogenic change, many species have suffered major habitat and population losses (4, 5), which are expected to have major consequences for current and future genetic diversity (6–8). Without global assessments of current genetic diversity across species, setting reasonable genetic diversity protection targets remains difficult. While direct genetic DNA sequencing provides precise measurements of species’ genetic health, it remains expensive and difficult to scale across many species. As a result, global conservation efforts have focused on demographic proxies for genetic diversity. These are based on the biological insight that the number of individuals within a species and its geographic distribution correlate with genetic diversity (9, 10). Animal species with rapid declines in their habitat or small population sizes often show lower levels of genetic diversity (9, 11, 12). Existing proxy indicators come from three sources: First, the International Union for Conservation of Nature (IUCN) Red List uses various criteria thresholds of population declines or geographic range reduction to classify thousands of species into different threat categories. Second, the Living Planet Index (LPI) monitors vertebrate populations through various censuses since the 1970s (13). Third, a new headline indicator approved in the Kunming-Montreal UN CBD COP15 tracks whether populations have sufficient breeding individuals to maintain genetic diversity (e.g., effective population size, *N_e_*, is greater than 500). A complementary indicator reports on between-population genetic diversity by monitoring the fraction of populations that are maintained within a species (14). Populations may hold unique traits and genetic adaptations that support species survival. However, the relationship between population size (or number of populations) and genetic diversity is complex, since genetic diversity is influenced by both spatial structure and temporal population dynamics. While proxy indicators represent an important first step in developing scalable metrics, critical gaps remain in translating demographic proxies into quantitative estimates of genetic diversity loss in present and future timescales, particularly for setting global biodiversity protection targets in the 21st century.

To bridge this gap, we recently described the mutations–area relationship (MAR) (6). MAR relates one measure of genetic diversity, the allelic richness, *M*, to the geographic area inhabited by a species, *A*, via a power law:$M\propto A^{z_{\mathrm{MAR}}}$. The relationship between allelic richness and geographic area is controlled by a scaling parameter (*z_MAR_*) that characterizes population structure (6, 15). Classic population genetic models (16) assume a randomly mating population, where *M ∝* log(*N*), which can explain diversity within a population. In contrast, MAR incorporates the effects of continuous population structure via the *z_MAR_* parameter, allowing it to capture how genetic diversity changes between-populations, as multiple populations may hold unique genetic diversity. With knowledge of the *z_MAR_* parameter and habitat area loss of a species, conservation practitioners can use MAR to estimate the percentage of genetic diversity lost. In previous work, we applied MAR across many species using estimates of habitat loss and various demographic proxies from conservation indices, resulting in our previous estimate that over 10% of genetic diversity has already been lost (6). (See *SI Appendix, Text S4* for more in-depth background on MAR.)

While MAR can be readily applied to translate demographic proxies into genetic diversity estimates for global assessments, in practice, genetic diversity loss is far more complex. There are multiple metrics to characterize genetic diversity, but MAR was developed and tested for only one metric of genetic diversity, allelic richness. It is unknown how other metrics, such as nucleotide diversity π (hereafter π diversity) that is also broadly used in population genetics, scale with area. While allelic richness is known to be highly sensitive to population disturbances (17, 18), π diversity may better reflect long-term genetic health and adaptive potential (19). Additionally, MAR provides only a static description of allelic richness immediately following habitat loss. It fails to model dynamic processes occurring in natural populations, such as increased genetic loss in more fragmented and isolated populations or the cumulative impact of genetic drift over time.

To address these limitations, we developed spatiotemporal models to understand how habitat loss patterns impact genetic diversity over time. We analyzed our models in the context of two main types of habitat loss patterns—edge contraction and fragmentation—finding that the shape of habitat loss impacts both short- and long-term genetic diversity dynamics. Given the complexity of diversity loss patterns, we tested whether power laws could capture main trends in our model predictions. We found that short-term π genetic diversity loss fits a power law relationship with area—a pattern we analogously coin the genetic diversity–area relationship (GDAR). Finally, we analyzed data from 29 plant and animal species to obtain realistic parameter values for our GDAR and spatiotemporal modeling frameworks. Using these parameters as guides, we estimated past, present, and future genetic diversity loss by translating demographic proxies of 4,611 species from the IUCN Red List, LPI, and GBF Indicator 2 datasets.

## Results

### A Spatiotemporal Modeling Framework of Genetic Diversity.

To address the challenge of predicting π diversity loss from demographic proxies, we built population genetics numerical methods and simulations that can model complex habitat loss scenarios. Our modeling framework considers a species distributed across a landscape of interconnected habitat patches, with individuals migrating among patches. Using this framework, we model the dynamics of genetic diversity loss across a gradient of habitat loss and population isolation levels under two habitat loss scenarios. We then developed theoretical machinery to compute how genetic diversity changes over time after patches are removed from the habitat (hereafter termed *WFmoments*). This work differs from existing approaches in three ways. First, this framework explicitly models arbitrarily complex spatial population structures present in real species (i.e., from one dimension receivers, to any two or three dimension habitats with any complex connections). Second, it models realistic landscape-level habitat loss scenarios that are more relevant to conservation efforts rather than modeling a simple bottleneck in a panmictic homogeneous population (20, 21) (i.e., spatial extinction fronts, fragmentation, etc.). Third, it models genetic diversity dynamics, tracking nonequilibrium π diversity over time, rather than just the initial and final equilibria points.

In brief, *WFmoments* considers the classic multideme Wright–Fisher (WF) population genetics model, where the habitat of a species is represented as a set of connected populations or habitat patches (22). Within each habitat patch (i.e., deme), the population is randomly mating, but complex spatial structure arises because individuals can migrate between patches at specified rates. Habitat loss is modeled by removing one or more habitat patches from the model. The key innovation of *WFmoments* is its efficient numerical machinery for computing how genetic diversity changes over time under this well-established model. (**SI Appendix*, Mathematical Appendix*.)

**Fig. 1.**
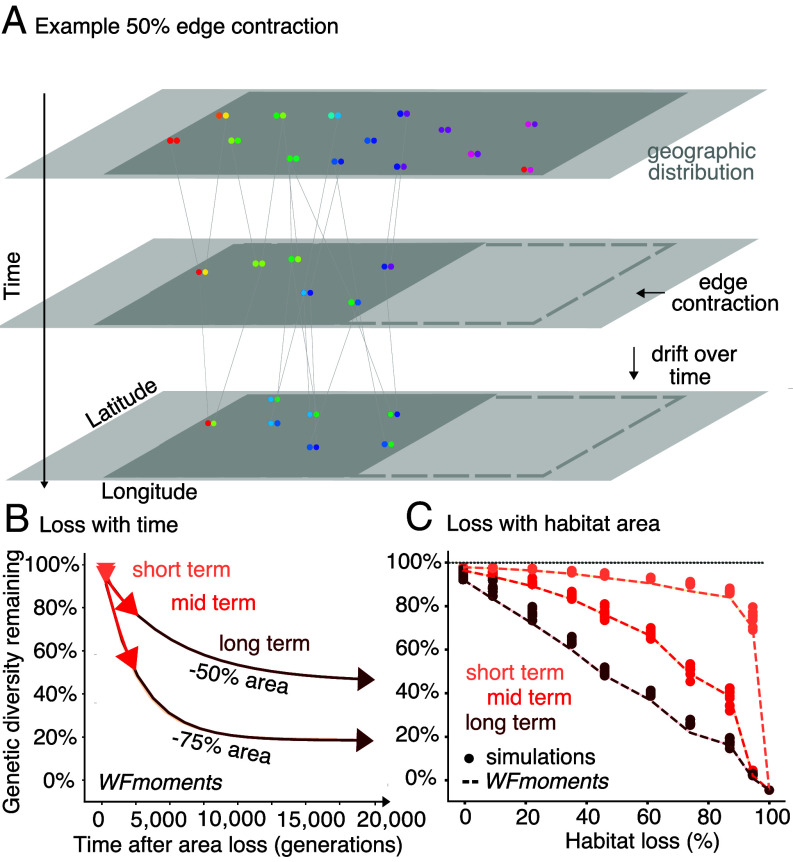
Genetic diversity after habitat destruction (*A*) Cartoon depicting a geographic distribution of a diploid species with spatial genetic structure (colors), the genealogy of genotypes, and how genetic diversity changes after area or population losses through edge contraction. (*B*) Genetic diversity continues to decrease over time. Trajectories were tracked across 20,000 generations at 50% and 75% habitat loss. Arrows represent average genetic diversity projections based on our *WFmoments* framework. Pink, red, and maroon arrows represent average short-term, mid-term, and long-term genetic diversity projections (Species parameters: F_ST_ = 0.3 and θ = 10^−4^). (C) Genetic diversity, π, decreases with increasing habitat percentage loss. Overlay of *WFmoments* (dotted lines) projections and simulation (dots) of population genetic diversity with increasing habitat loss.

In parallel, we validated the predictions made by *WFmoments* through a more biologically realistic simulation-based framework using SLiM v4.1 (23). We modeled non-WF dynamics, which are intractable in theoretical models, with individuals distributed on a continuous geographic landscape. The models incorporate specific dispersal rates, competition, spatially dependent mating, and an age structured population (*Methods Summary*, Fig. 1, and *SI Appendix, Text S1*). Throughout this paper, we simulated landscapes starting with 5,000 individuals that freely roam and reproduce. The number of individuals fluctuates over the course of simulations, but is maintained near 5,000 individuals by setting a carrying capacity for the landscape. Patterns hold for larger populations, which is key to understanding genetic diversity loss in broadly distributed species not only threatened isolated species (**SI Appendix*, Supplemental Materials*). Our simulations contain a long burn-in phase, ensuring that the population has reached equilibrium, as assessed by the stability of genetic diversity over time (*SI Appendix*, Fig. S20). In order to simulate habitat loss, we set the local carrying capacity of regions in the habitat to zero, making them uninhabitable (Fig. 1). This directly scales the total number of individuals that can be supported by the habitat. For example, a 50% habitat reduction sets the carrying capacity to zero for half of the habitat, resulting in a landscape that can only support approximately 2,500 individuals and hence results in a population roughly half the original size (*Methods Summary*).

### Minimal Short-term Genetic Diversity Loss Following Edge Contraction.

To understand the impact of habitat loss on genetic diversity, we began by modeling habitat loss that occurs along one edge of the habitat range, hereon termed “edge contraction” (Fig. 1). Utilizing both *WFmoments* and SLiM, we modeled genetic diversity trajectories from the time of habitat loss until genetic diversity re-equilibrated. In order to compare different habitat loss scenarios, we report genetic diversity metrics at three timepoints: one generation after habitat loss (short term), at the new equilibrium (long term), and at an intermediate timepoint (mid term).

We found that short-term loss of genetic diversity increases with the amount of habitat lost in a predictable manner (simulation results predicted by *WFmoments*: R^2^ = 0.98, *P* < 1 × 10^−16^, n = 81, Fig. 1*C*). Yet, the proportion of genetic diversity lost is substantially less than the proportion of habitat lost, with the precise relationship depending on the population structure of the species (*SI Appendix*, Fig. S3). In particular, the short-term loss of π diversity occurs faster in species with more spatial structure for a given amount of habitat lost (*SI Appendix*, Fig. S2). For instance, with 50% habitat loss, a species with high rates of migration and hence essentially no population structure (average F_ST_ across every pair of demes ≈ 0) experiences only a 4.7% (95% CI across *WFmoments* predictions = 4.5 to 4.9%) short-term π diversity loss (*SI Appendix*, Fig. S3). In contrast, a species with limited migration and hence stronger population structure (average pairwise F_ST_ ≈ 0.9) experiences a higher short-term π diversity loss of 9% (95% CI across *WFmoments* predictions = 7.9 to 10.0%) (*SI Appendix*, Fig. S3). This is expected, as species with strong population structure contain genetic diversity heterogeneously distributed between-populations, compared to species without population structure, where diversity is held within-populations. These short-term genetic diversity losses hold across landscapes of different dimensions (e.g., a one-dimensional landscape such as a river system, *SI Appendix*, Fig. S22 and *Text S2*) and whether habitat loss occurs instantaneously or as a gradual decline over time (*SI Appendix*, Fig. S1), suggesting that the relationship between habitat loss and short-term π diversity loss under edge contraction is robust.

### Genetic Diversity Continues to Decrease in the Long Term Under Edge Contraction.

We next quantified the magnitude of genetic diversity loss over time after population or habitat decline, as we expected that genetic drift will continue to erode diversity. Following classic population genetics theory, one might assume that within a single panmictic population (i.e., no population structure), the expected equilibrium π diversity should be directly proportional to the effective population size (24, 25): *E*[*π*] = 4*N*_*e*_ *μ.* For instance, if *N*_*e*_ represents area or population abundance (if population size is proportional to area), a 50% reduction in population size would proportionally reduce *π* diversity by 50% in the long term. However, this theory assumes stable, well-mixed populations—conditions that are often violated in spatially structured species—and that a long-term equilibrium is reachable. As a result, it is unclear how π diversity will behave over time in complex nonequilibrium dynamics following habitat loss. To address this question, we used *WFmoments* and simulations to model the dynamics of π diversity from the time at which habitat loss occurred until the population re-equilibrated (Fig. 1 and *SI Appendix*, Figs. S1 and S4). We found that similar to short-term π diversity estimates, population structure largely determined the relationship between π diversity loss and habitat loss. As expected for a species with no population structure (average pairwise F_ST_ ≈ 0), the long-term equilibrium closely follows the classic expectation: the proportion of π diversity lost is nearly equal to the proportion of habitat lost (*SI Appendix*, Fig. S3). A similar trend was observed for allelic richness (*SI Appendix*, Fig. S15). In contrast, for a species with strong population structure (average pairwise F_ST_ = 0.9), a ~50% habitat loss results in a lower long-term π diversity loss of only ~27% (*SI Appendix*, Fig. S3). Interestingly, while increasing population structure led to larger short-term π diversity losses, long-term genetic diversity loss is less severe in more structured populations (*SI Appendix*, Fig. S3, see theoretical interpretation in *SI Appendix, Text S3*).

Long-term genetic diversity losses are calculated at the time of re-equilibration, which is too far in the future to be relevant for conservation policies, and so we wanted to understand genetic diversity loss on a policy-relevant timescale. We found that genetic diversity decays exponentially over time (Fig. 1*B*, theoretical explanation in Supplementary *SI Appendix, Text S3*), with the majority of long-term losses occurring within the first *N_e_*/2 generations—i.e. substantially lagging after habitat loss (26). For instance, for a weakly structured population of size *N_c_* = 5,000 experiencing 50% habitat loss, we find that genetic diversity decreases from 100% to ~79% within the first ~2,200 generations before reaching a final equilibrium of ~50% genetic diversity over ~20,000 generations (Fig. 1*B*). As such, we define the “mid term” to be *N_e_*/2 generations following habitat loss in Fig. 1*C*.

To put these time points into the context of the GBF’s 2050 goals for biodiversity, species with short generation times and small populations will be the most at risk of losing π diversity from drift following habitat loss. For instance, the federally endangered Miami blue butterfly, with an estimated population size (*N_c_*) of ~100 (27) and ~3 to 4 generations a year, could reach what we call the mid term within 50 generations or 12.5 y (assuming *N_c_* = *N_e_*; although *N_e_* is often smaller hence the dynamics are likely faster). In contrast, for species with large populations and slower generation times such as large vertebrate mammals or long-lived trees, our short-term estimates may be more reflective of genetic diversity in 2050.

Overall, our findings highlight a worrisome point: given the weak impact of habitat loss on immediate short-term π diversity loss, species may not currently show clear signs of genetic diversity loss (28). Nevertheless, since habitat loss profoundly impacts mid-term and long-term π diversity, populations could already be on a trend of genetic diversity loss for decades or centuries to come. In order to avoid more dramatic long-term losses, drastic actions to restore habitat area and population sizes within species need to be taken.

### Genetic Diversity Recovery Is Slow Following Habitat Restoration.

Our observation that genetic diversity decreases slowly raises the critical question of how quickly genetic diversity may be recovered by restoring habitat. Using our SLiM framework, we simulated two scenarios: a habitat recovery (where populations naturally colonize the recovered habitat) and population restoration (where individuals across the intact landscape are actively translocated to the restored habitat) (*SI Appendix*, Fig. S23 and *Text S5*). In both cases, our population genetic framework shows that even if habitats are restored, π diversity is slow to react and recover to original levels (*SI Appendix*, Fig. S24 and S25). For short-lived species, recovery may be faster but for slow growing species, recovery of genetic diversity may be unlikely within the century. These results have significant implications for GBF Target 2, which aims to restore 30% of degraded ecosystems by 2030. Our fundamental conclusion is that the buildup of genetic diversity through new mutations is incredibly slow, as previously shown in (24), emphasizing the importance of preventing habitat destruction in the first place.

### Genetic Diversity Dynamics Under Complex Landscapes with Fragmentation.

**Fig. 2.**
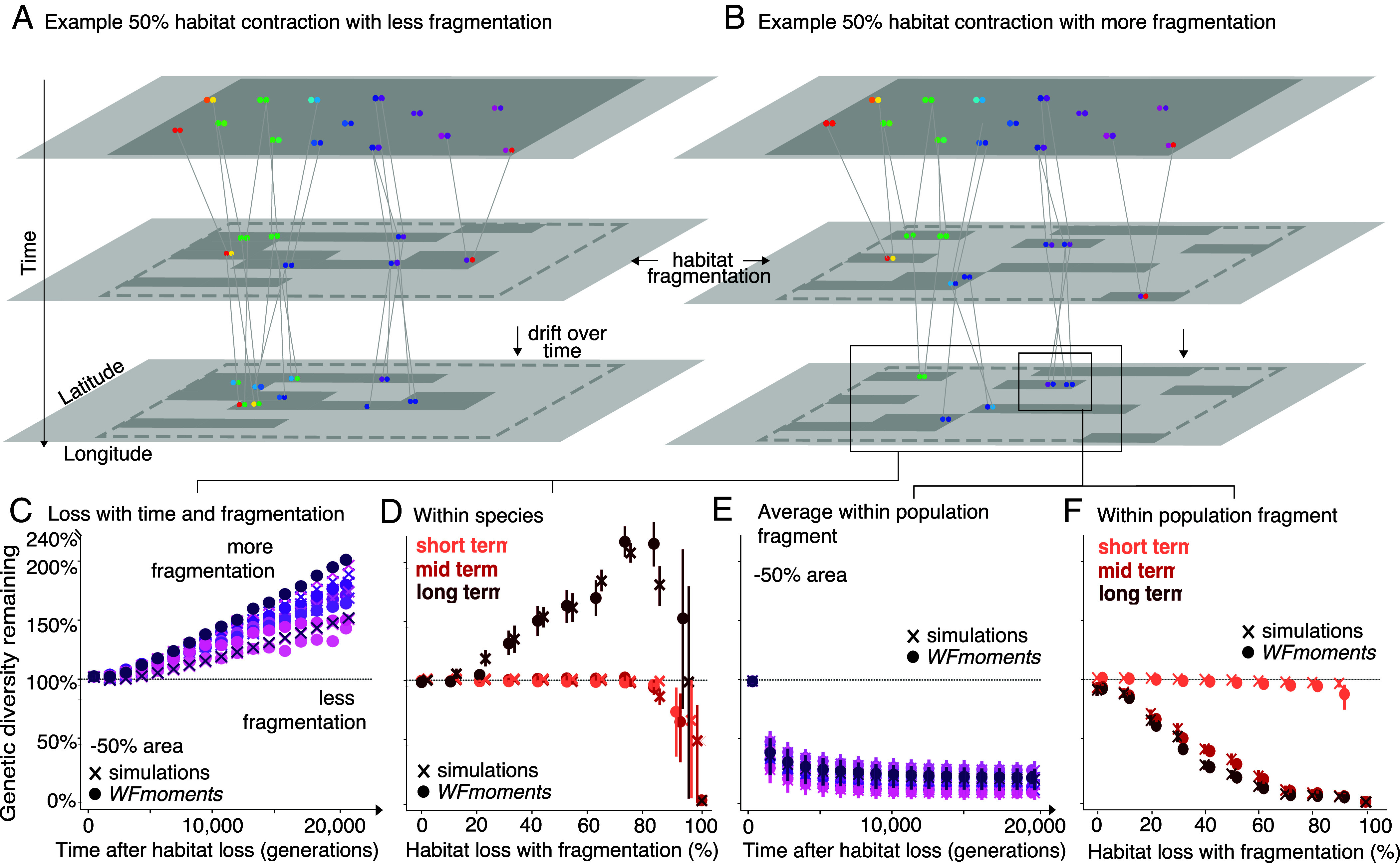
Impacts from habitat destruction by fragmentation are only detectable in within-population diversity. Cartoon depicting a geographic distribution of a diploid species with spatial genetic structure in genetic diversity (colors), the genealogy of genotypes, and how genetic diversity changes after area or population losses under 50% habitat loss with (*A*) less fragmentation or (*B*) more fragmentation. Habitat fragmentation maps vary according to level of connectivity between patches of landscape. (*C*) Species-level genetic diversity increases with time and level of fragmentation. *WFmoments* projections of genetic diversity loss (π) due to 50% habitat loss over 20,000 generations. Each point-series represents a different 50% fragmentation map, where each map is colored by whether it is more (dark purple) or less (light purple) fragmented, as seen in (*A*) and (*B*). Species-wide genetic diversity, π_species_, increases over time with fragmentation. (*D*) Overlay of *WFmoments* (dots) and simulation-based averages (cross) of short-, mid- and long-term genetic diversity loss (π) due to habitat loss with habitat fragmentation (averages of 121 random fragmentation maps). Each cross represents an average measure of π across a landscape per simulation overlaid with its 95% interquantile range of short- (pink), mid- (red), and long-term (maroon) genetic diversity. (*E*) Within-population genetic diversity under habitat fragmentation decreases with time. Within-population genetic diversity, π_local_, across 20,000 generations for the same *WFmoments* projections as (*C*). (*F*) Within-population genetic diversity under habitat fragmentation decreases with increasing habitat loss. Within-population genetic diversity, π_local_, across increasing habitat loss for the same *WFmoments* and simulation projections as (*D*).

So far we have focused on habitat loss by edge contraction, removing habitat grids from one edge of the habitat, which leaves the remaining habitat well-connected. However, land use changes could instead lead to fragmented habitats (29). Thus, we used SLiM and *WFmoments* to better understand how the dynamics of π diversity depend on the pattern of habitat fragmentation, by partitioning the species landscape into 100 equally sized habitat patches and creating habitat fragmentation. To simulate fragmentation, we stochastically removed these habitat patches (Fig. 2 *A* and *B* and *SI Appendix*, Fig. S7). Across 121 simulations, we created landscapes of different degrees of fragmentation. Habitats of low fragmentation still maintain well-connected patches (Fig. 2*A*), while habitats of high fragmentation have more isolated patches with little to no gene flow (Fig. 2*B*). Similar to the case of edge contraction, our simulations showed that π diversity is highly insensitive to habitat loss in the short-term (Fig. 2 *C* and *D*). However, unlike edge contraction, π diversity counterintuitively increases in the long-term under high fragmentation scenarios (Fig. 2*D*). For example, at ~90% habitat loss with fragmentation, π diversity increased by 263% (range: 100 to 263%; also observed in allelic richness, *SI Appendix*, Fig. S14). As habitats get fragmented, populations may become isolated. Over time, isolated populations diverge and become more distinct from each other, leading to overall high genetic diversity across habitat patches (*SI Appendix*, Fig. S14). This phenomenon has been previously documented in classic population genetics and is captured by both *F-*statistics and the Wahlund effect (30, 31). However, the observed inflation is not unpredictable: provided with the same simulated fragmented landscapes, *WFmoments* was also able to recapitulate increased π diversity patterns observed in simulations (R^2^ = 0.69, *P* < 1 × 10^−16^, n = 121 Fig. 2*C* and *SI Appendix*, Fig. S22). We therefore tested whether landscape metrics such as the scale of fragmentation or habitat connectivity could help explain these counterintuitive genetic diversity trajectories.

We tested the relationship between the level of fragmentation (i.e., more or less isolation of patches) and the inflation magnitude of long-term π diversity across 121 independent simulations. We found several landscape ecology metrics (32) that significantly explained those inflations: including total core area, which captures all connected habitat patches (R^2^ = 0.76, *P* = 1.62 × 10^−25^), spatial connectedness, which captures how well habitat patches are linked across the landscape (R^2^ = 0.87, *P* = 1.11 × 10^−35^), the general perimeter of patches, which captures the total amount of habitat edge or boundary (R^2^ = 0.53, *P* = 2.49 × 10^−14^), and patch and edge density, which captures the fragmentation level or number of distinct habitat patches per unit area (R^2^ = 0.63, *P* = 2.12 × 10^−18^) (Fig. 2*C* and *SI Appendix*, Figs. S10 and S11). Our results suggest that for practitioners studying impacted species or populations in complex landscapes, a quantitative understanding of the pattern of landscape fragmentation will be needed to interpret species-wide genetic diversity trends.

While π diversity increases following habitat fragmentation when considering the species as a whole, some of this is driven by an increase in divergence across the population, potentially masking a reduction in the amount of genetic diversity within subregions of the habitat. As such, we wanted to understand whether local measures of genetic diversity (hereon termed π_local_ diversity) would reveal genetic diversity loss following habitat fragmentation. Using our continuous space fragmentation simulations (Fig. 2), we calculated π_local_ diversity by computing π using only individuals falling within a single grid cell (1/100th of the landscape) that we moved along the landscape and averaged the resulting local values of π. We consistently found that π_local_ diversity decreases across habitat fragmentation scenarios (Fig. 2*E*). We then wanted to explore how π_local_ diversity was affected by the amount of habitat lost. Overall, we detected a small but significant reduction in the short term (linear regression *b* = −0.09, *P* = 2.57 × 10^−236^, R^2^ = 0.30, Fig. 2*F* and *SI Appendix*, Fig. S14), followed by a sharp mid-term reduction in π_local_ diversity (linear regression *b* = −1.20, *P* < 1.0 × 10^−16^, R^2^ = 0.69, Fig. 2*F* and *SI Appendix*, Fig. S14) and an even larger long-term reduction (linear regression *b* = −1.26, *P* < 1.0 × 10^−16^, R^2^ = 0.72, Fig. 2*F* and *SI Appendix*, Fig. S14). Similar, but more substantial trends hold when using within-population allelic richness as the genetic diversity metric (*SI Appendix*, Figs. S14 and S15).

Our findings have key conservation implications. Genetic diversity protection goals and indicators within the GBF must include explicit guidelines on which genetic diversity metrics to use and how they are calculated. Critically, these metrics need to account for population structure. As seen from our findings, aggregating individuals from discrete, isolated populations may result in misleading π diversity values (e.g., a large species-wide π but very low diversity within any given patch) and hence, result in misleading conclusions about species’ genetic health.

### Power Laws Can Predict Short-term Genetic Diversity Loss Following Edge Contraction.

While our simulations and *WFmoments* provide detailed insights into how π diversity is impacted by area loss and complex fragmentations, simple and easy-to-use tools could enable more rapid forecasting for conservation practitioners. As such, we wanted to see if short-term π diversity losses could be well-modeled by a power law. In prior work, we posited that the relationship between short-term allelic richness loss, *M_lost_*, and habitat loss, *A_lost_,* can be well-approximated by a simple power law: *M_lost_* $=1-(1-{A_{\mathrm{lost}})}^{z_{\mathrm{MAR}}}$ which we called the MAR. While MAR can be used to forecast short-term changes in allelic richness, it would be ideal to also have simple approximations for π diversity. To address this, we fitted the power laws to our simulations and *WFmoments* predictions and found that short-term π diversity loss, π*_lost_*, follows a power-law relationship (accuracy for power law fit to theory and simulations: R^2^ = 0.96): π*_lost_* = $1-{\left(1-A_{\mathrm{lost}}\right)}^{z_{\mathrm{GDAR}}}$, where the exponent, *z_GDAR_*, also depends on the strength of population structure. We term this the genetic diversity–area relationship (GDAR) (*SI Appendix*, Fig. S2 and Tables S1–S4). However, GDAR is inherently limited to predicting only short-term π diversity (Table 1). In order to also facilitate easy access to mid- and long-term genetic diversity estimates, we provide *WFmoments* precomputed tables of π diversity loss forecasts (*SI Appendix, WFmoments approximation table*). With these tables, practitioners can look-up combinations of F_ST_ values and habitat area losses (with/without fragmentation) to find the expected value of π diversity loss over time. We believe that GDAR and *WFmoments* have complementary use cases and both will be useful in making predictions about π diversity (Table 1).

**Table 1.** Summary of modeling frameworks to translate demographic proxies to genetic diversity loss

Method	Scenario	Input needed	Advantages	Limitations
MAR	Short-term allelic richness	Area loss, *z_MAR_* population structure parameter	Easy to use and scalable	Limited to short-term estimates of allelic richness under edge contraction
GDAR	Short-term π diversity	Area loss, *z_GDAR_* population structure parameter	Easy to use and scalable	Limited to short-term estimates of π diversity under edge contraction
*WFmoments* & approximation tables	Short-/mid-/long-term and π diversity	Area loss, population structure via *F_ST_*, population size, migration rates.	Arbitrarily complex population sizes, habitat geometries, and migration rates	Requires specifying models with a python API, and computation can take minutes. Approximation tables for precomputed scenarios solve this
SLiM simulations	Short-/mid-/long-term allelic richness and π diversity	Population and genetic parameters (population size, migration, reproductive type, density effects, landscape properties, natural selection)	Enables almost any degree of population biology and genetic complexity, Graphical User Interface (GUI) available, excellent documentation	Requires scripting; computationally demanding for large simulations.

We describe the scenarios that each of the methods can predict, and their advantages and limitations.

### Translating Demographic Proxies Indicators into Genetic Diversity Loss Percentage Across Species.

To demonstrate the utility of our methods, we translated current GBF indicators into global genetic diversity loss percentage estimates resulting from land use change and habitat or population decline. Although precise species-specific data on historical geographic area reductions are scarce for many species, GBF indicators serving as demographic and threat proxies have been widely documented across thousands of species. We focused on three key indicators: the IUCN Red List—which established threshold values for geographic area losses over the past three generations or 10 y (*SI Appendix*, Table S20); the LPI—which documents population census sizes at two or more timepoints since the 1970s; and the KM-GBF Ne 500 headline indicator and complementary indicator—which jointly reports the proportion of populations maintained within a species.

**Fig. 3.**
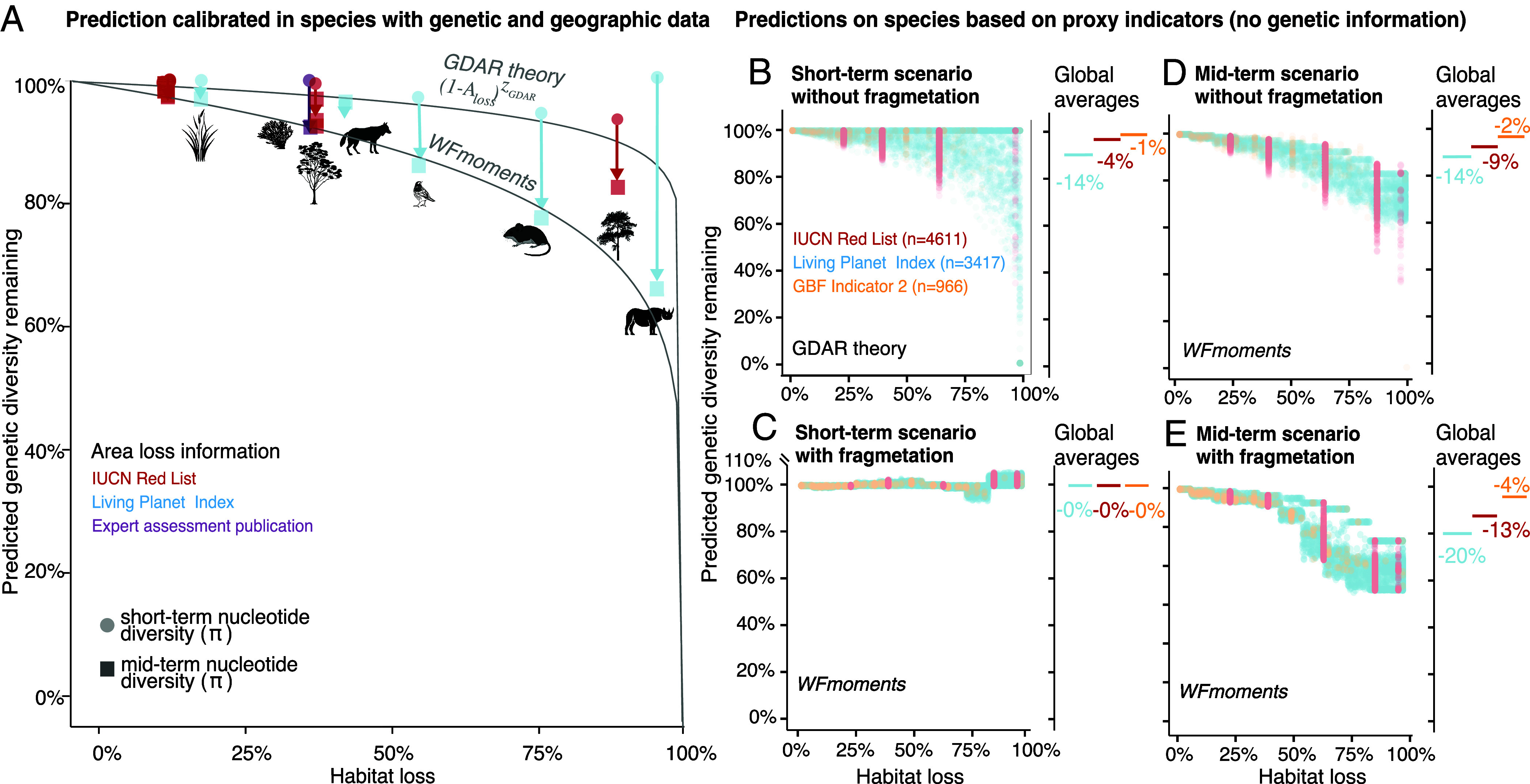
Estimates of genetic diversity loss across species from conservation indicators in short- and mid-term, and contraction and fragmentation scenarios. (*A*) Estimates of genetic diversity loss for 29 plant and animal species with genomic data to calibrate GDAR/*WFmoments* predictions. Using species-specific F_ST_ or *z_GDAR_* obtained from ADMIXTURE (36) and *mar* v0.0.3 (35), we predict short-term π diversity losses (dots). Using *WFmoments,* we also predict mid-term π diversity losses (squares). Area or population losses per species are extracted from Red List category (red), Living Planet Index (blue), or expert-annotated ranges (purple). Predictions follow edge contraction expectations. (*B*) Short-term π genetic diversity loss projections for 4,611 species with recent area or population loss information from Red List (red), LPI (blue), or KM-GBF complimentary indicator 2 (orange). GDAR predictions of short-term π diversity use sampled *z_GDAR_* parameters following the empirical distribution from species in (*A*). (*C*) Short-term π diversity loss projections for the same 4,611 species listed in (*B*), assuming area loss with high fragmentation (*WFmoments* predictions). (*D*) Mid-term π diversity loss projections for the same 4,611 species in (*B*), assuming area and population losses stop after short-term reduction. *WFmoments* predictions of π diversity use randomly sampled F_ST_ values following the empirical distribution from (*A*). (*E*) Mid-term π diversity projections for the same 4,611 species, assuming area loss with high fragmentation and isolation (*WFmoments* predictions). Next to the predictions across all species and scenarios (*B*–*E*), global arithmetic averages are reported.

First, we predicted genetic diversity loss for the 4,611 plant, animal, and fungal species in the IUCN Red List that contain criteria on habitat or population loss (*SI Appendix*, Table S20). The IUCN Red List documents population size or area of occupancy declines over at least 10 y or three generations under criteria A2–A4 (6, 33, 34). We used these declines as proxies for habitat loss in our GDAR and *WFmoments* frameworks by assuming habitat size and population sizes are proportional (*SI Appendix*, Fig. S18 and Table S20). For each species, we made three types of genetic diversity predictions. First, short-term π diversity loss under edge contraction using GDAR. Second, short-term π diversity loss under fragmentation using *WFmoments*; and third, mid-term π diversity loss using *WFmoments*. To make predictions using GDAR, we needed estimates of the power law exponent, *z_GDAR_*. We estimated *z_GDAR_* by first simulating extinction across the 29 plant and animal species with genomic data using the *extinctionsim* module in *mar* v0.0.3 (35), yielding a mean *z_GDAR_ =* 0.03, range *=* [0.00 to 0.45] (mean power-law fit R^2^= 0.677, range = 0.10 to 0.98, Fig. 3*A* and *SI Appendix*, Table S18). For example, the endemic California tree *Pinus torreyana,* for which we have genome-wide data, has likely lost over 80% of its habitat based on its critically endangered status (Fig. 3*A* and *SI Appendix*, Fig. S17 and Table S19, see Red List entry: https://www.iucnredlist.org/species/42424/2979186). With an inferred *z_GDAR_* of 0.028, we predict its short-term expected π diversity loss to be at least 1 − (1- 0.8)^0.02809^ = 4.4% (Fig. 3*A*). To make predictions over time using *WFmoments,* we needed species-specific F_ST_ values. We estimated F_ST_ by running ADMIXTURE (36) on the same 29 species with genomic data, yielding a mean F_ST_ = 0.26, range = [0.01 to 0.7]. Using our *WFmoments* precomputed loss tables (*SI Appendix*), for example, the common Australian tree *Eucalyptus melliodora,* with an average F_ST_ = 0.01, would incur a mid-term loss of 6.3% given its estimated 30% habitat loss (Inter-Quantile Range [IQR] = 5.2 to 7.3%). *E. melliodora* is a species with low population structure and hence we expect fewer losses compared to species with increased spatial heterogeneity (Fig. 3*A*). For the 4,611 Red List species, we sampled *z_GDAR_* and F_ST_ values from the empirical distribution obtained from our 29 species with genetic data (Fig. 3 *A*–*E* and *SI Appendix*, Table S20). Applying these approaches across all species in the Red List, we find an overall average short-term π diversity loss of 4.5% (IQR = 0 to 6%) (Fig. 3*B*). Assuming no further habitat losses in the future, we predict that genetic diversity will continue to be lost resulting in an average loss of 9.5% (IQR = 3.4 to 13.1%) π diversity in the mid term (Fig. 3*E*).

Next, we used demographic survey data across 5,579 threatened and nonthreatened vertebrate species from the LPI (37). While there are a number of approaches to analyze the LPI time series data as discussed in literature (38), we focused on a straightforward approach, taking the earliest and the latest observation per species to compute a fraction decline (*N_present_/N_past_*) for each species, which resulted in a mean 64% population loss (IQR = 40 to 93%) (*SI Appendix*, Fig. S16). We applied the same approach to these species as we did for the IUCN Red List species, using this estimate of population loss as our proxy for habitat loss. Using GDAR, we estimate that across 4 decades of data, there is an average π diversity loss of 13.9% (IQR = 0 to 12.2%) (Fig. 3*B*). Mid-term projections with *WFmoments* estimate a 14.2% π diversity loss (IQR = 5.0 to 21.8%) (Fig. 3*E*).

Finally, the Group on Earth Observations Biodiversity Observation Network (GEOBON) Genetic Composition group showcased the feasibility of the 2022’s GBF genetic proxy indicators by carefully annotating 966 species for nine countries (39) (*SI Appendix*, Fig. S19). The fraction of wild populations lost per species (GBF complementary Indicator 2) averaged 18.6% (IQR = 0 to 35%). Among the remaining populations, 76% (IQR = 60 to 100%) had *N_e_* > 500 (GBF headline Indicator 1). Using the proportion of populations lost from GBF Indicator 2 as a proxy for proportion of habitable area lost in combination with GDAR, we estimated an average short-term π diversity loss of 1.3% (IQR = 0%) (Fig. 3*B*). Mid-term projections using *WFmoments* estimate a π diversity loss of 2.2% (IQR = 0 to 2.0%) (Fig. 3*E*). In principle, one could also use GBF’s Indicator 1 for population genetic predictions, but this approach presents challenges as *N_e_* within a population not only depends on the census population size but also on gene flow, migration, and connectivity between populations. For instance, increased gene flow increases local *N_e_* even when the census population size remains constant. Nonetheless, this qualitative indicator is helpful in prioritizing conservation efforts (40).

All of the predictions described above were made assuming habitat loss results from edge contraction (Fig. 2), but we expect fragmentation to limit genetic diversity loss predictability in certain scenarios. To explore how the shape of habitat loss impacts our forecasts, we also conducted predictions assuming that habitat loss occurred via fragmentation. Because we lacked quantitative connectivity metrics of populations evaluated in LPI, Red List, and new GEOBON-driven GBF indicators, we used *WFmoments* and assumed high levels of random fragmentation and isolation levels as in our simulations (Fig. 2). Since we observed that in fragmented landscapes π_local_ diversity may be more reflective of the genetic health of a species (Fig. 2 *E* and *F*), we made our forecasts using π_local_ diversity (Fig. 3 *C* and *F*). Across all three indicators, we show an average 0% π_local_ loss (IQR = 0 to 0.4%) in the short term and a 15.5% π_local_ loss (IQR = 4.1 to 27.5%) in the mid term. These genetic diversity losses will become more dramatic over the next decades even if no more populations or habitats are lost.

These estimates of current and future genetic diversity losses across thousands of species solidify our previous warnings of substantial genetic biodiversity declines in the Anthropocene (6). Many species have likely already started losing genetic diversity, even those with populations of *N_e_ >* 500. Even if these losses may be hard to measure empirically on short timescales (8, 28, 41), our work showcases the consequential accumulation of losses over time. This temporal perspective suggests that genetic conservation goals should be centered around targets that not only preserve near-term genetic diversity, such as the GBF’s 2050 goals for biodiversity, but also prioritizes the ability to maintain genetic diversity over time. For instance, the Swedish Agency for Marine and Water Management proposed that populations should retain at least 95% of their heterozygosity over 100 y (34). Data-driven and theoretically informed models of the spatiotemporal dynamics of genetic diversity are therefore essential to create genetic diversity loss scenarios for the 21st century. Our work underscores an urgent need for action and provides a tool to inform future biodiversity policy targets. It is crucial that we utilize this window of opportunity to ambitiously recover and reconnect populations, ensuring species’ long-term genetic protection.

## Methods Summary

### Forward Simulations Setup for Continuous 2D Space.

To study genetic diversity trajectories under habitat loss, we used SLiM v. 4.0.1 (23). We first simulated diploid genomes using a single, 10^8^ long chromosome, and set both recombination rate and mutation rate to 10^−8^. Unless otherwise stated, these parameters are kept constant for any downstream analysis or simulation scenarios. Using SLiM’s non-WF mode, we simulated a single population of size, *N_c_* = 5000. The spatial structure of our population is established by associating each individual with a continuous 2D coordinate (i.e., latitude and longitude). These coordinates are then used to govern four demographic processes: mate choice, reproduction, dispersal rate, and competition.

For mate choice and reproduction, if the focal individual is of fertile age, a mate is chosen randomly and weighted based on spatial proximity, to generate a number of offspring. The number of offspring is determined by sampling from a poisson distribution parameterized to control average fertility. Any newly generated offspring’s position is then drawn from a Gaussian distribution centered at the location of the focal individual with a SD of dispersal rate.

At the end of each SLiM simulation tick, all individuals disperse randomly at a rate that is twice the offspring dispersal rate. Spatial competition is implemented based on the local population density felt by each individual. This is established using a Gaussian distribution that governs the strength of spatial competition, which is rescaled based on the carrying capacity, *N_c_*. Finally, we scale an individual’s fitness nonlinearly with its age, up to a maximum age of 10 SLiM simulation ticks. Unless otherwise specified, the following parameters are kept constant for all our simulations: fertile age = 3, Poisson fertility rate = 0.5, dispersal rate = 0.05, offspring dispersal rate = 0.025, carrying capacity, *N_c_* = 5000. Note that the effective population size in population genetics, N_e_, is not a set parameter but an emergent parameter that may differ from census size *N_c_* in nonpanmictic, spatially structured populations with complex age structures, reproduction, and overlapping generations (all complexities that are expected in nature).

### Running Simulations on Large Populations Using SLiM and Msprime.

First, in order to ensure that enough time has passed for spatial population structure to develop, we run our simulations for 1,000,000 simulation ticks (~200,000 generations) (*SI Appendix*, Fig. S20). As predicted by the isolation by distance pattern (42), individuals sampled closely together in 2D space are now more genetically related than individuals sampled far apart. To reduce the major computational burden of simulating every mutation, we used tree sequence recording (43) to track the full genealogy of all individuals in the simulation which are either alive at the end of the simulation or sampled through time using the treeSeqRememberIndividuals function of SLiM. For the purposes of our simulation, we simulate 1,000,000 SLiM ticks (~200,000 generations) to establish spatial structure and ensure that all sampled individuals fully coalesce (in which case the value of genetic diversity resulting from these simulations would be at equilibrium and stable) (*SI Appendix*, Fig. S20).

In order to ensure computational efficiency, especially in simulating large populations of *N_c_* = 5,000, we simulated coalescence backward in time with msprime (43). This process has been referred to as “recapitation”(44), where an incomplete genealogy of a large population with multiple roots (from SLiM) is “recapitated” using coalescent simulation backward in time. This is made possible by using the tree sequence data structure to record and simulate genealogies using both SLiM and msprime. Since we are only concerned with how processes such as dispersal affect neutral variation across space and through time, we can use the “recapitated” tree sequence to overlay mutations onto the full genealogy of all sampled individuals. The rationale is that under neutrality, mutations do not affect the structure of the genealogy. Therefore, we can first simulate the genealogy without mutations, before overlaying neutral mutations to reduce computational burden. We then extracted the resulting genotypes of all individuals from the tree sequence for downstream analysis.

### Genetic Diversity Metrics.

Genetic diversity is measured as π, the average pairwise difference between all possible pairs of individuals, and S, allelic richness (or the number of segregating sites). In addition, we proceed to calculate both within-population (one grid cell) and species-wide metrics of both π and S. We measured genetic diversity using pairwise genetic diversity with the equation below, where n is the number of remaining individuals, L is the total number of SNPs selected, and p_i_ is the allele frequency at genomic location i. The percentage of area loss was calculated by dividing the total number of habitat cells removed with the number of total habitat cells constructed from all geo-referenced individuals at each iteration. We measured genetic diversity as π, the average nucleotide diversity, calculated using the standard formula incorporating sample size (n), sequence length (L), and allele frequencies (p_i_): π = (n/n − 1) (1/L) Σ_i_^L^ 2p_i_(1 − p_i_).

#### Within-population genetic diversity metrics.

Within-population genetic diversity metrics calculate the average pairwise difference between all possible pairs of individuals (π_local_) within each grid cell across all 100 grid cells. In total, we obtain 100 values of π or S, and proceed to calculate the average of π or allelic richness across all 100 grid cells. This ensures that individuals that belong in the same population are compared with each other.

#### Species-wide genetic diversity metrics.

Species-wide metrics (π_species_) are calculated by getting the average pairwise difference between all possible pairs of individuals within all grids, so individuals–possibly in separate grids–are compared to each other to obtain an average value for all 100 grid cells.

### Simulating Different Scenarios of Human Impacts on Species Habitats.

We partitioned the continuous space simulation landscape in SLiM into a 10 ⨯ 10 grid. During habitat reduction, we removed grids to reduce the simulation landscape and scale the carrying capacity by a function of habitat size to ensure that a 50% reduction of habitat also leads to a 50% reduction in carrying capacity of the entire habitat. For instance, a 50% reduction would imply a removal of 50 grids and a reduction of carrying capacity, *N_c_* = 2,500. For each grid, we calculated both the pairwise genetic diversity, π diversity, and number of segregating sites (S) between individuals across the entire habitat. Our simulations included two ways of habitat destruction, edge contraction, and habitat loss with fragmentation.

#### Instantaneous range contraction simulations.

To assess the dynamics of genetic diversity in populations undergoing habitat loss, we performed edge contraction simulations starting from 10% habitat loss to 90% habitat loss at 10% increments, with nine replicates at each progression of habitat loss. Under edge contraction, we performed habitat loss simulations by removing grids from one edge, in order to ensure that the remaining habitat remains as one continuous landscape. Each simulation was run for 1,000,000 SLiM simulation ticks before instantaneous habitat loss occurred at 1,000,001 SLiM time points. After which, we tracked the dynamics of both π diversity and allelic richness in the short-term (at 1,000,001 SLiM simulation ticks after habitat loss), in the mid-term (at 1,010,025 SLiM simulation ticks or approximately *N_e_*/2 or ~2,200 generations after habitat loss), and in the long-term (at 1,080,025 SLiM simulation ticks or approximately ~20,000 generations after habitat loss).

Under habitat loss with fragmentation, we performed habitat loss simulations from 6 to 93%. We utilized a random integer generator to select which grids in our 10 ⨯ 10 simulation map to “extinct,” hence causing the range of habitat loss percentages to be variable. Each simulation run generates a unique habitat fragmentation map. In total, we simulated 121 different habitat loss maps with fragmentation.

In order to briefly explore other habitat fragmentation scenarios, we performed habitat loss simulations on a larger geographical grid of 20 ⨯ 20. Utilizing a random integer generator to select grids in our 20 ⨯ 20 simulation map to “extinct,” this simulation setup ensures that habitat loss is distributed more evenly across the geographical grid and at smaller areas than previously implemented. We term this as “mini” fragmentation to understand how genetic diversity changes with time if fragmentation occurs at much smaller scales across a wider geographical range.

#### Altering parameters to study spatial population dynamics.

Given the expectation that key population genetic parameters affect the dynamics of how genetic diversity changes with habitat loss, we wanted to explore how population size and migration rate might affect π diversity estimates under various habitat loss scenarios. We utilized population sizes ranging from 500 to 10,000 (exceptionally 20,000 individuals for computational efficiency) and varied dispersal rate respectively to test our variable Fst values to mimic low, medium, and high population structure. Specifics of combinations of population sizes and dispersal rates can be found listed in the legend of *SI Appendix*, Fig S1.

#### Gradual habitat loss simulations.

To explore alternative habitat loss scenarios, we also looked at gradual habitat loss dynamics where habitat loss would decrease by 1% every 11 generations. This simulation was run for 2,000 SLiM time points before gradual habitat extinction of 50% habitat loss which occurred from 2,001 SLiM timepoints (approximately 450 generations) to 2,250 SLiM timepoints (500 generations).

### *WFmoments* Intuition Beyond Classic Population Genetics Theory.

Existing approaches for understanding the effect of habitat loss on genetic diversity either ignore spatial structure in populations (45); ignore the effect of new mutations (46); do not model temporal dynamics; or are purely phenomenological (6, 47) making it difficult to interpret the relationship between genetic diversity and habitat loss in terms of population genetics forces (i.e., drift, mutation, and migration). We developed *WFmoments* to remedy these shortcomings.

*WFmoments* is based on a multideme Wright Fisher (WF) model, where individuals are grouped into locally panmictic “demes,” and individuals can migrate from one deme to another, allowing for the encoding of arbitrary spatial connectivity patterns. As input to *WFmoments*, one specifies a habitat by providing a number of demes and the rate of migration between each pair of demes. For example, one could specify demes arrayed in a two-dimensional grid with migration only between adjacent demes to approximate a two-dimensional spatial habitat with free migration across the space. Then, given population sizes for each deme (approximating the density of individuals across space) and a mutation rate, *WFmoments* calculates the equilibrium level of π for any possible combination of demes (e.g., π within a particular deme, or a species-wide π). One can then specify changes to the habitat (e.g., destruction of one or more demes, changes in migration rates) or species (e.g., changing population sizes, changing mutation rates) and track how π (in any possible combination of demes) changes over time.

The key to *WFmoments* is the observation that π in any combination of demes only depends on the first two moments of the joint distribution of allele frequencies across demes. Under the neutral WF model, how these moments change over time forms a closed system of ordinary differential equations that can efficiently be exactly solved using tools from numerical linear algebra. See *SI Appendix*, *Mathematical Appendix* for more details.

### Fitting *WFmoments* Models to Simulations.

Our modeling (see *SI Appendix*, *Mathematical Appendix*) has three free parameters: effective population size, mutation rate, and migration rate. Under standard WF dynamics these may all be derived from real-world observable data, namely the census population size, rate of new mutations per generation, and number of migrants per generation. Yet, our simulations contain a number of features that strongly deviate from the standard WF model. There are overlapping generations, individuals live in continuous space instead of discrete demes, and there is density-dependent selection and spatially controlled mate choice. The WF model is surprisingly robust to deviations from its assumptions with the caveat that parameters must be interpreted in terms of an “effective population size” that depend on these model deviations in complex ways (48). As a result, we expect our mathematical model to be able to recapitulate key features of the simulation, albeit with modified parameters.

To find the parameters that provided a good fit between our theory and our simulations for Figs. 1 and 2, we used least-squares fitting. We approximated the continuous habitat in the simulations by a 10 by 10 grid of square demes, with migration between adjacent demes. We then fixed the effective population size and optimized the least-squares fit between the theoretically predicted species-wide π from simulations and theory over three parameters: the mutation rate, the migration rate between adjacent demes, and a time-scaling for converting units of time in our theoretical model to SLiM timepoints in the simulations. Optimization was performed using the “Powell” method in scipy.optimize (49–51).

### Reanalyses of Population Genomic Datasets of 29 Species.

#### Generating F_st_ values across diverse species.

We utilized datasets across diverse species that were collected for refs. 6 and 52. All datasets were transformed into PLINK files using PLINK v1.9 (53). For additional information on data processing, refer to the Supplementary materials of ref. 6. For computational efficiency, we generated F_st_ values using admixture v1.3.0 (36), specifically utilizing the command `admixture –cv sample.bed <K or number-of-clusters>` where we tested a range of 1 to 15 K and picked the K with the lowest cross-validation (CV) error. We reported the average and maximum F_st_ values across K populations for each species (*SI Appendix*, Table S19).

#### Simulating short-term extinction in empirical datasets.

To confirm the genetic extinction patterns we observed in population genomic simulations and mathematical models, we also simulated short-term extinctions in empirical sequencing datasets of 29 wild plant and animal species [detailed description of datasets available in (6)]. Briefly, for each species, the empirical dataset contains geo-referenced individuals broadly sampled across its geographical distribution and naturally occurring mutations were discovered through various DNA sequencing methods. We conducted the analyses with up to 10,000 randomly selected biallelic single nucleotide polymorphisms (SNPs) for each species sampled genome-wide, or in the largest chromosome for those species with large genomes. We sought to use unfiltered SNP datasets to avoid ascertainment biases.

For each species, the in silico extinction simulations were conducted by iteratively removing map cells in the sample map, and geo-referenced individuals falling within the removed cells are considered extinct. Genetic diversity was estimated from the genotype matrix of remaining individuals using the R package MAR v0.0.3 (35). We measured genetic diversity using pairwise genetic diversity with the equation below, where n is the number of remaining individuals, L is the total number of SNPs selected, and p_i_ is the allele frequency at genomic location i. The percentage of area loss was calculated by dividing the total number of map cells removed with the number of total map cells constructed from all geo-referenced individuals at each iteration. Genetic diversity was estimated as π = (n/n − 1) (1/L) Σ_i_^L^ 2p_i_(1 − p_i_).

We implemented two hypothesized patterns of extinction to match the population genomic simulations presented in the main text (*SI Appendix*, Table S5 and S6). The random scheme involved extinctions occurring randomly across the range as would be expected by the habitat fragmentation scenario, while the south-north scheme had extinctions starting in the north and moving southward simulating the impacts of climate change. Each species underwent 20 replicates for both the random and south-north extinction schemes.

### Estimating Global Genetic Diversity Loss Based on Global Population and Area Loss Census.

#### LPI.

In order to estimate global genetic diversity loss, we extracted population sizes of 5,230 species that have been tracked for over 3 decades via the LPI 2024 (37). For each species, we summarized the population data in a few ways. To obtain the fraction of populations lost, we obtained the arithmetic mean of populations over three decades. For more information on the distribution of LPI data used, *SI Appendix*, Fig. S16. Given complexities in measuring population declines using time-series data (38), we opted to calculate the average arithmetic abundance decline from earliest to latest census (100 × *N_present_ / N_past_* + 1) across populations within a species. Finally, we subsetted the LPI data to estimate genetic diversity loss for only species that experienced a decline in average arithmetic abundance across populations. This left us with 3,417 species. We then utilize population decline values as a proxy for habitat loss.

We assumed that F_st_ across the LPI species would follow a normal distribution with mean 0.270 and SD 0.211 (as determined by the F_st_ values we obtained across diverse species, *SI Appendix*, Table S19). Utilizing these F_st_ and habitat loss approximations, we estimated the amount of short-, mid- and long-term genetic diversity utilizing both our MAR/GDAR and *WFmoments* theoretical framework (*SI Appendix*). To obtain an average global genetic diversity loss estimate, we summed the total genetic diversity loss to obtain the total amount of genetic diversity loss divided by all populations.

#### Red list.

We first obtained criteria of area loss for all categories from the Red List database (www.iucnredlist.org). Counts of each category and criteria used in Red List classification are summarized (*SI Appendix*). We filtered the species in the IUCN Red List to only those that have data corresponding to criteria A2, A3, or A4, which left us with 4,611 species. Genetic diversity loss values were then calculated for these 4,611 species across the seven Red List categories: Extinct, Likely Extinct, Critically endangered, Endangered, Vulnerable, Near Threatened and Least Concern. We approximated area loss by using population size loss criteria for each of the six categories by taking the arithmetic mean of the minimum and maximum of the range. For example, a species categorized as critically endangered when population size loss is between 80 to 95%. Utilizing these mean area loss values for each category, we then used MAR/GDAR and *WFmoments* to make predictions of short-, mid- and long-term genetic diversity π (*SI Appendix*).

## Supplementary Material

Appendix 01 (PDF)

## Data Availability

Scripts; Spatial simulation scripts, *WFmoments* scripts, visualization scripts and data have been deposited in Zenodo [DOI: https://doi.org/10.5281/zenodo.18603464 (54); https://zenodo.org/records/18761464 (55)]. All other data are included in the article and/or *SI Appendix*.
